# The effect of home exercise on ovulation induction using clomiphene citrate in overweight underserved women with polycystic ovarian syndrome

**DOI:** 10.1186/s40834-016-0025-2

**Published:** 2016-08-24

**Authors:** Jodi Nagelberg, Heather Burks, Sara Mucowski, Donna Shoupe

**Affiliations:** 1 Department of Obstetrics and Gynecology, Los Angeles, CA 90033 USA; 2 Keck School of Medicine, Los Angeles, CA 90033 USA; 3 University of Southern California, Los Angeles, CA 90033 USA; 4 Department of Obstetrics and Gynecology, Keck School of Medicine, Health Sciences Campus, IRD 530, Los Angeles, CA 90089 USA

**Keywords:** Pregnancy Rate, Thyroid Stimulate Hormone, Ovulation Induction, Underserved Population, PCOS Woman

## Abstract

**Background:**

Age-adjusted rates of obesity are reported to be 35.8 % among US adult women and 49 % in some race/ethnicity, underserved populations. (1). Underserved populations often have less access to weight-loss intervention options and are at high risk for obesity related problems including anovulation, infertility, pregnancy-related complications and adverse long-term health outcomes. (2). The purpose of this study was to evaluate a home exercise plan using a pedometer on weight loss, ovulation induction and pregnancy rates in our overweight and obese underserved clinic population.

**Methods:**

Twenty one overweight (BMI ≥ 25–29.9) and obese I-II (BMI ≥ 30–39.9) 18–42 years old were recruited. Participants received an exercise/nutrition questionnaire at the initiation and completion and called weekly for 4 weeks. Ten participants were randomly assigned to the home exercise program (PedGp). PedGp received a pedometer, daily step-count goal, and were called to increase goal by 50 % weekly. All participants then underwent clomiphene stair-step ovulation induction. All study participants were referred to the University Wellness Clinic for diet and exercise counseling.

**Results:**

There were high percentages of women with co-morbidities in both groups including fatty liver, low vitamin D, hyperlipidemia, hypothyroidism, prediabetes and diabetes.

1. Those completing the 4-week home program increased baseline steps by 21.2 % weekly. Only 3/10 women reached at least one weekly goal of 50 % increase. Although the goal was rarely met, participants who completed study had increased number of daily steps.

2. Greater number in PedGp lost weight or stayed the same (5/10 vs. 2/11).

3. Greater number in PedGp spontaneously ovulated (4/10 vs. 1/11) or became pregnant (4/10 vs. 3/11). (not statistically significant due to small sample size).

**Conclusion:**

There are high percentages of comorbidities in this population. Although the goal was rarely met, participants who completed study had increased number of daily steps. A greater number in PedGp lost weight or stayed the same. A greater number in PedGp spontaneously ovulated or became pregnant (not statistically significant due to small sample size). Importantly, 40 % of women who lost weight became pregnant. This is highly encouraging and suggests that the development of pedometer interventions may prove a cost effective option. *Weight loss programs for this population hold promise and efficient hospital or community-based programs may prove beneficial.*

## Background

As obesity rates continue to rise across the US, more patients seeking fertility services are overweight or obese. In 2012, 35.8 % of adult US women are categorized as obese (BMI ≥ 30 kg/m^2^), and this increases to up to 49 % in some racial, underserved populations [1]. Within this setting, many of these patients have polycystic ovarian syndrome, which contributes to their infertility. Further, these populations are at high risk for obesity related problems including infertility, anovulation, pregnancy-related complications and adverse long-term health outcomes [2]. Unfortunately, there is limited evidence-based data for the management of these populations in a non-IVF setting.

First line treatment for anovulatory PCOS women seeking fertility is ovulation induction, historically, via clomiphene citrate, often with the use of metformin [3]. It is generally believed that achieving ovulation induction in PCOS patients is more difficult than in non-PCOS populations. Further, higher BMI values are also associated with poorer outcomes.

Weight loss serves as the best means of physiologically inducing ovulation in overweight and obese women with PCOS [4]. Prior studies have shown the anthropometric benefits of weight loss in these populations, with regard to restoring ovulatory cycles and allowing spontaneous pregnancy [5]. Specifically, with loss of only 5-10 % of body weight, studies have shown resumption of ovulation within 6 months [6]. However, achieving weight loss in these studies often involves rigid dietary restrictions or expensive, time consuming exercise interventions, less accessible to underserved populations [7]. In contrast, the pedometer is an inexpensive, simple tool that may motivate and monitor exercise, and be used as a surrogate to reliably and objectively quantify physical activity [8, 9].

We set out to evaluate the effect of a home exercise regimen using a pedometer plus nutrition counseling on weight loss, ovulation induction and pregnancy rates, in underserved, overweight or obese PCOS women desiring fertility. It was hypothesized that those in the pedometer group will have increased rates of ovulation and pregnancy as compared to those without the intervention.

## Methods

### Study subjects and procedures

This study was conducted at Los Angeles County + University of Southern California (LAC + USC) Medical Center. Per the 2013 California Office of Statewide Health Planning and Development (OSHPD) census, Hispanics constitute over 60 % of the population in the surrounding Medical Service Study Area (MSSA), with 68 % of the population determined to be within 200 % of the federal poverty. Further, nearly two-thirds of the hospital’s payer mix includes Medicaid and uninsured or under-insured populations. Study participants were recruited from the Reproductive Endocrinology (REI) Clinic who were seeking fertility evaluation and treatment. Those with a diagnosis of PCOS per the NIH criteria were eligible for enrollment if they were female of reproductive age (18–42 years), anovulatory with a BMI categorized as overweight or obese, and without other known conditions contributing to infertility, including hyperprolactinemia, hypothyroidism, male factor or uncontrolled insulin resistance. Patients with untreated endometrial hyperplasia, undiagnosed endometrial bleeding, uncontrolled hypertension or diabetes or thyroid dysfunction were excluded from the study [10]. Diabetes and prediabetes were diagnosed in accordance with the American Diabetes Association criteria. Patients with hemoglobin A1c (HbA1c) levels between 5.7 and 6.4 % were considered to be pre-diabetic [11]. Subjects were further screened to exclude those with a functional cyst greater than 15 mm, candidates for intrauterine insemination due to an HIV positive male partner, or other medical conditions precluding an advisable pregnancy. Recruitment continued through a 12-month recruitment period. Written informed consent, approved by the University of Southern California Health Science Campus Institutional Review Board, of each participant was obtained prior to inclusion in the study. Upon determination of eligibility, demographics, baseline lab results, information regarding each ovulation induction cycle and prior pregnancy outcomes were abstracted from each participant’s medical record.

### Study design

In this randomized controlled pilot study, ovulation and pregnancy rates in overweight and obese PCOS women receiving a pedometer were compared to those not receiving pedometers. All study participants were treated according to the usual established LAC + USC REI clinic protocols, which includes various blood draws for analysis. All study participants were referred per protocol to the Wellness Clinic for dietary and exercise counseling.

All participants received a validated exercise/nutrition questionnaire to determine their baseline diet and exercise habits. After completion of the questionnaire, participants were randomized to either receive a pedometer or no pedometer, according to an even (pedometer) or odd (no pedometer) outcome via a random number generator, and all data was recorded by these assigned codes. Pedometers were used according to package insert instructions. Those who were assigned to the intervention group also received an exercise diary in which to record their pedometer reading. All participants were called each week to discuss their exercise and nutrition goals. Counseling was the same in each group, except those in the pedometer group also reviewed their weekly step count and were given specific goals to increase the number of steps by 50 %, with an ultimate goal of 10,000 steps per day. After 4 weeks, all participants’ weights were recorded at their visit.

Following the intervention period, the current guidelines and clinic protocols for stepwise ovulation induction with clomiphene citrate was followed for all participants. In the event of pregnancy at any time point, the patient was referred to OB and their chart was periodically reviewed to ascertain pregnancy outcome.

### Statistical analysis

Significance of baseline HbA1c, glucose levels, thyroid stimulating hormone (TSH), testosterone and Vitamin D levels were compared in those who ovulated or became pregnant via independent t-tests. Categorical variables were compared between groups using Fisher Exact and Chi-Squared tests.

## Results

Twenty-one overweight (BMI ≥ 25–29.9) and obese I-II (BMI ≥ 30–39.9) 18–42 year-old PCOS women seeking infertility services were recruited and met inclusion criteria. Ten were randomly assigned to receive a pedometer and 11 to the non-intervention group. Table 1 shows the baseline metabolic markers of the included participants. No significant differences were seen (p >0.05) amongst BMI, HbA1c, glucose, hypothyroid status, testosterone, DHEAS or Vitamin D levels between the control and pedometer groups. There was a high percentage of comorbidities in this underserved population, including low Vitamin D, overt and subclinical hypothyroidism, non-alcoholic fatty liver, hyperlipidemia, elevated androgens, hypertension and depression, diabetes and prediabetes.

**Table 1:** Baseline metabolic markers

	Pedometer (*n* = 10)	No Pedometer (*n* = 11)
BMI ≥ 25–29.9 kg/m^2^	30 % (*n* = 3)	63.6 % (*n* = 7)
BMI ≥ 30–39.9 kg/m^2^	70 % (*n* = 7)	36.4 % (*n* = 4)
HbA1c ≥ 6 %	30 % (*n* = 3)	18.1 % (*n* = 2)
Glucose ≥ 100 mg/dL	40 % (*n* = 4)	45.5 % (*n* = 5)
Hypothyroidism	30 % (*n* = 3)	36.4 % (*n* = 4)
Elevated Testosterone (≥49 ng/dL)	20 % (*n* = 2)	63.6 % (*n* = 7)
Elevated DHEAS (>320 μg/dL)	20 % (*n* = 2)	27.3 % (*n* = 3)
Vitamin D, 25-OH deficiency (<30 ng/mL)	70 % (*n* = 7)	63.6 % (*n* = 7)

Data on ovulation and pregnancy rates in participants relative to baseline characteristics is seen in Table 2. A significant association was found between BMI and ovulation, (*p* = 0.048, SD 3.39). There were no significant relationships between BMI and pregnancy or HbA1c, elevated glucose, TSH, testosterone, or low Vitamin D and ovulation or pregnancy rates.

**Table 2:** Baseline mean ± SD indices and *p* values in ONLY participants with ovulation or pregnancy

		Ovulation	Pregnancy
BMI	31.41 (3.39)	0.048*	0.18
HbA1c	5.90 (1.60)	0.46	0.33
Glucose	106.52 (50.39)	0.54	0.59
TSH	2.45 (0.87)	0.35	0.31
Testosterone	47.62 (17.93)	0.29	0.33
Vit D Deficiency	27.68 (12.28)	0.78	0.57

* Significant at *p* < 0.05 level compared to non-responders

A total of 7 patients became pregnant, 4 in the pedometer group and 3 in the control, although this was not statistically significant. A greater number in pedometer group spontaneously ovulated (4/10 vs. 1/11) and became pregnant (4/10 vs. 3/11), although this was not statistically significant due to the small sample size. 40 % of the patients in the pedometer group completed the 4-week home program and increased their baseline steps by 21.2 % weekly. Further, 5 of 10 (50 %) of the participants in the pedometer group lost weight or stayed the same, compared to 2 of 11 (18.2 %) of those without a pedometer [*p* = 0.5]. The step count goal of a 50 % increase in steps weekly, to a maximum of 10,000 steps per day was rarely met (in 3 of 10 participants), although participants who completed the study had an increased number of daily steps.

## Discussion

PCOS is often associated with anovulatory cycles, and therefore infertility. As obesity rates in the US continue to trend upward, an increasing number of women seeking out infertility services, including those with PCOS, will fall within an elevated BMI range [1]. While first line treatment for PCOS women with infertility is ovulation induction with clomiphene citrate, this syndrome is associated with poorer ovulation induction outcomes than in those without the condition. Further, obesity is independently correlated with the interruption of regular ovulatory cycles, while a 5-10 % weight reduction is associated with return of ovulatory function [5, 6]. Assisted Reproductive Technologies (ART), including IVF, remains a viable option, although it is largely unsubsidized by insurance carriers, necessitating alternative, more cost-effective fertility options. Such is the case for the women with PCOS in the LAC + USC REI underserved clinic population. This study evaluated the effectiveness of one such economical option, by randomly assigning PCOS women to receive a pedometer or not, to ascertain the efficacy of an at home exercise plan and nutritional counseling on weight loss, ovulation induction and pregnancy rates.

The results of this study are promising, as a greater number in the pedometer group lost weight or stayed the same (5/10 vs. 2/11), spontaneously ovulated (4/10 vs. 1/11), or became pregnant (4/10 vs. 3/11) as compared to the control arm. Those who completed the 4-week exercise regimen had a 21.2 % increase in step count from baseline, although often without meeting the 50 % increase step count goal. Further, 40 % of women who lost weight became pregnant, suggesting a similar study with a larger sample size and increased compliance may find a significant increase in these study endpoints. Since the weekly follow-up phone calls proved to be a highly time intensive interventional structure, alternative protocols may be more feasible in a community setting. In motivated participants, increasing daily physical activity, as objectively measured by a pedometer recording daily steps, may result in weight loss and increased ovulation and pregnancy rates.

Various factors serve as unique hurdles against the effectiveness of an at-home intervention in underserved populations. Firstly, there is a high percentage of comorbidities in this population. Although patients with known unregulated comorbidities contributing to infertility were excluded, many of the participants had conditions known to independently affect fertility, such as thyroid dysfunction. Further, while the use of pedometers has been proven to be a reliable and objective measurement of physical activity, and one that may encourage exercise, social factors may have prevented the pedometer-assigned participants from achieving their weekly step count goals or consuming low calorie foods [8, 9]. Per patient reporting, these include the safety of their neighborhoods, long work hours and the cost and accessibility to fresh produce, amongst others. Thus, one must consider the effectiveness of a pedometer-based intervention in this clinic population, given their specific obstacles.

This study investigated an area with a current paucity of evidence-based data, through the use of a low-cost intervention. Possible limitations of the study include a small sample size, poor compliance and lack of sufficient power to identify statistically significant differences in baseline characteristics and ovulation and pregnancy rates amongst the pedometer and control groups.

While the limited sample size precludes one from defining the definitive effectiveness of such an intervention, the use of a pedometer in an at-home exercise regimen with weekly step-count goals and phone follow-up, may prove to be a cost-effective intervention for both weight loss and fertility. Future research may further elucidate the relationship between pedometer use weight loss, ovulation and pregnancy rates, in addition to identifying additional cost-effective options.
